# Protective Multifunctional Fibrous Systems Based on Natural Fibers and Metal Oxide Nanoparticles

**DOI:** 10.3390/polym13162654

**Published:** 2021-08-10

**Authors:** Joana C. Araújo, Raul Fangueiro, Diana P. Ferreira

**Affiliations:** 1 Centre for Textile Science and Technology (2C2T), University of Minho, 4710-057 Guimarães, Portugal; rfangueiro@dem.uminho.pt (R.F.); diana.ferreira@det.uminho.pt (D.P.F.); 2 Department of Mechanical Engineering, University of Minho, 4710-057 Guimarães, Portugal

**Keywords:** advanced protection, CWAs, BWAs, multifunctional fibrous structures, metal oxide nanoparticles, natural fibers

## Abstract

In recent years, an unprecedented increase in the development of products and technologies to protect the human being has been observed. Now, more than ever, the world population is exposed to several threats, harmful to their well-being and health. Chemical and biological hazardous agents stand out as one of the biggest threats, not only for the military forces, but also for the civilians. Consequently, it’s essential to develop personal protective systems that are able to protect their user, not only passively, but actively, being able to detect, adsorb, degrade and decontaminate pesticides, pollutants, microorganisms and most importantly: chemical/biological warfare agents. One recent strategy for the development of active fibrous structures with improved functions and new properties is their functionalization with nanoparticles (NPs), especially metal oxides. Although their known effectiveness in the decomposition of harmful agents, the NPs could also include other functionalities in the same structure using low quantities of material, without adding extra weight, which is of huge importance for a soldier in the battlefield. The use of natural fibers as the substrate is also very interesting, since this material is a much sustainable alternative when compared to synthetic ones, also providing excellent properties.

## 1. Introduction

Protection is essential not only to maintain the safety of professionals that are exposed every day to several dangers, but also for the general population [1]. It can be said that today’s world is full of threats responsible for several impactful and harmful effects that not only directly affect the human being, but that are also very dangerous to the natural environment. Most recently, the COVID-19 pandemic reminded us of the variety of agents that can strongly influence public health, becoming one of the majors concerns for governments all over the world, being object of a large monetary investment [2,3].

We are more and more exposed to harmful threats, namely ultraviolet (UV) radiation, pathogenic microorganisms, ionizing and nonionizing radiations, burning fire hazards, between others. Thus, the development of personal protective systems, that represent an effective barrier between the user and these noxious agents, which include chemical agents and also biological ones is of huge importance, and has become one of the main concerns for governments worldwide [4].

CBRN (Chemical, Biological, Radiological and Nuclear) warfare agents are truly a threat for the human being. The first reported use of harmful chemical and biological agents goes back to ancient Greek and Roman times. However, it was in the 19th century that this type of incidents became more frequent. The first use of chemical agents as weapons of mass destruction was in World War I, representing the beginning of not only the research, but also the use of microorganisms in warfare [5]. Other agents are also an increasing problem for the worldwide population. The sum of several factors like, increasing urbanization and population, the excessive use of non-renewable resources and over exploration of the natural ones and especially, the fast industrialization, is responsible for a wide range of several damages to the environment [6]. It’s well known that the majority of industries, from a broad array of areas, generate organic pollutants that represent a serious and dangerous threat, not only to the environment, but also to the public health [7].

Thus, the disposal of noxious pollutants without proper treatment, especially organophosphorus compounds, can be considered one of the biggest causes of environmental pollution and for the disruption of the society’s well-being [8]. Moreover, this type of compounds can be compared to neurotoxic chemical warfare agents (CWAs) since they also inhibit the Acetylcholinesterase (AChE) enzymatic activity. This inhibition causes harmful effects on the human being and can even cause death. Organophosphorus compounds are toxic materials that can be found in both pesticides and CWAs. Pesticides are considered less lethal than chemical weapons, but, in high quantities can lead to comparable effects. In fact, the World Health Organization (WHO) reports about three million cases per year of intoxication with organophosphorus pesticides [9].

Most of the already available protective systems, against this specific type of agents, are passive. Usually, these systems are developed only to act as adsorbent agents and are not capable of promoting the complete elimination of the harmful compounds through decontamination processes. Therefore, the development of fibrous structures that are protective, reactive and responsive takes special importance. These structures should be capable of self-decontamination as well as detection of a wide range of toxic materials [10]. One of the most recent trends in textile industry, that can also be one great strategy for the development of advanced protective fibrous structures, is the development of multifunctional products [11] based on their functionalization with nanoparticles (NPs), especially metallic and metal oxide ones. Due to their nanoscale and large surface area, the incorporation of NPs into textiles can provide several new functionalities, including the degradation of chemical/biological harmful agents [12]. Considering the environmental growing consciousness, the use of natural fibers for the development of personal protective textile structures is desirable over synthetic ones, because of their biodegradability, biocompatibility, low-weight, high abundance and low-cost. Thus, the functionalization of this type of fibers with NPs is also a very interesting and sustainable approach in this area [13].

## 2. Personal Protective Equipment

Personal protective equipment (PPE) refers to protective clothing, helmets, gloves, face shields, goggles, facemasks and/or respirators or other equipment designed to protect the wearer from injury or the spread of infection or illness [10]. In the specific case of protective clothing, which is considered one of the main categories of technical textiles, the main goal is to protect the user and to have functional properties, instead of aesthetical ones. Personal protective textiles can be classified in different categories depending on their applications, industrial, agricultural, military, civilian, medical, sports and space protective textiles. These materials are responsible not only for the user’s protection against outer threats, namely chemical, thermal, mechanical, biological and radiation, but also for the user’s comfort [1]. Thus, protective textiles need to present several properties like UV protection, antimicrobial activity, flame retardancy, decomposition of chemical agents, antistatic properties, monitoring/sensing behaviour, wound healing characteristics, hydrophobicity, between others. Nevertheless, if various properties are simultaneously present on the same structure, its applicability range can be considerably improved [14].

In order to choose the best materials to be used for specific situations, several factors need to be taken into consideration. Besides the nature of the threat in question, the weight, comfort, the level of protection and the duration of the necessary protection are very important [15]. Between all of the possible threats, CWAs and biological warfare agents (BWAs) are now considered one of the most critical issues [1].

### 2.1. Chemical Warfare Agents: Incidents and Classification

The use of CBRN harmful materials has been reported since ancient Greek and Roman times, even though their impact was not as relevant as today, due to the lack of knowledge related to this theme. It was in the 19th century, with the great advances in chemistry and in the chemical industry, that critical events caused by hazardous chemicals became more common. The use of chemical materials as weapons of mass destruction in World War I represented the start of the incessant search for more CWAs, as well as for the improvement of their efficiency [5].

The most impactful event related to a chemical agent was the 1995 Tokyo subway sarin gas attack, which resulted in 13 deaths and about 6300 casualties. This incident was the second sarin attack in Japan, being that just a year earlier, another attack on the city of Matsumoto, that killed 7 people and injured over 200, was performed. The same religious cult, Aum Shinrikyo, was the responsible for the two events [16].

The specific case of deliberate CBRN incidents against the civilian population by terrorist groups reveals the affected countries’ lack of preparation to deal with this type of events. Although incidents of large-scale rarely occur, an effective and fast emergency response is necessary since the consequences of these occurrences can be of great magnitude and escalate quickly [17].

**Figure 1:**
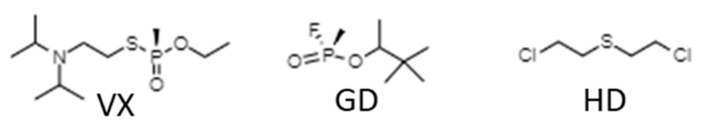
Molecular structures of the most cited CWAs in literature, VX, GD e HD.

**Figure 2:**
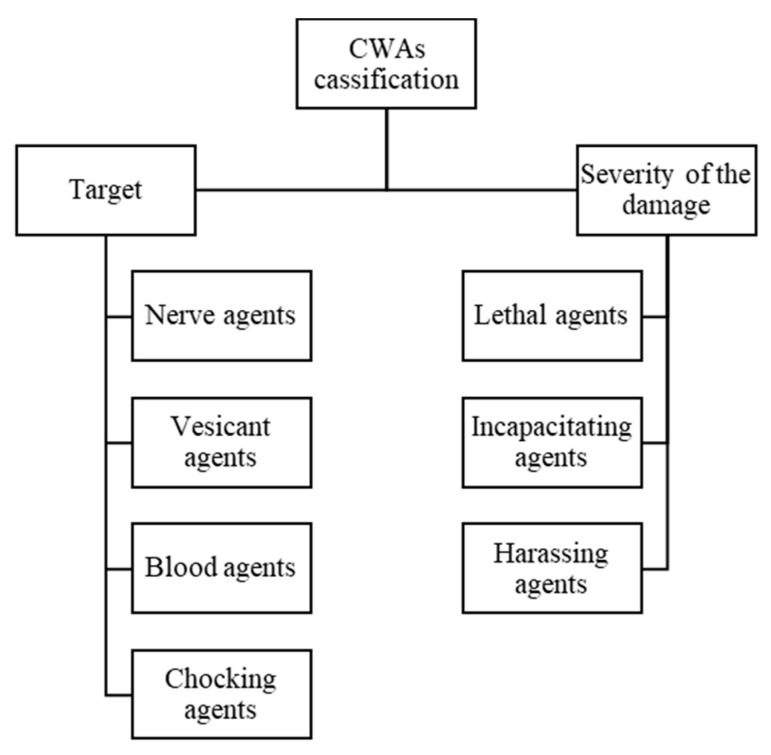
Classification of CWAs depending on their target and the severity of the damage.

CWAs are usually synthetical or natural derived chemical compounds that have the capacity to easily and quickly penetrate the skin, being in most cases incapacitating and even lethal [18]. These types of weapons are probably one of the most dangerous threats created by man, that can easily become agents of mass destruction. This group consists of CWAs that can be use in several forms and that are classified in four categories, according with their target. Some of the CWAs most cited in literature are VX, GD or soman, HD or mustard (Figure 1). They can be classified as nerve, vesicants, blood and chocking agents (Figure 2) [19,20].

Nerve agents (or neurotoxic agents): target the central nervous system, reacting with AchE enzyme, resulting in convulsions, respiratory collapse and even death [19]. Between these agents, the ones stand out are tabun, sarin, soman and VX [9]. They represent one of the most dangerous type of CWAs since they provoke fast effects and can enter the victim’s body by several routes [21]. For example, their skin permeability rates are of approximately 0.1 cm/min [18]. In the specific cases of the chemically pure forms of G-agents, like soman and sarin, they have a water-like appearance and are soluble in water and organic solvents. They are also volatile which makes the respiratory tract the most probable entryway in the victim’s organism [21].

Vesicants agents: lead to blister formation, attacking skin and mucous membranes (mainly the lungs) [19]. The most impactful and well-known vesicant agents are the mustard gases (ClCH_2_CH_2_XCH_2_CH_2_Cl, X=O, S, NR). Although on most occasions these types of agents are not lethal, they can strongly incapacitate the victims, which can result in hospitalization that can go from one to four weeks [9]. Their skin permeability rates are of approximately 2.0 cm/min [18].

Blood agents: are mainly cyanides and cause symptoms like choking of breath, vomiting blood, between others [19]. These compounds release cyanine ions in the organism and disturb the typical functions of the body, preventing the normal consumption of oxygen by the body tissues. The main agents in this category are hydrogen cyanide (HCN) and cyanogen chloride (CNCl) [22].

Choking agents (or pulmonary agents): lead to choking of breath since they attack the respiratory tract. In this group, the most important compounds are phosgene (COCl_2_) and diphosgene (Cl_3_COCOCl) [19,21].

CWAs can also be classified depending on the severity of the damage, since all of them are developed in order to cause acute harm, but some of them are not deadly. Thus, they can be divided in three categories, lethal agents, incapacitating agents and harassing agents (Figure 2) [23].

Lethal agents are the ones who cause death (at sufficiently high exposures). Some of them kill after only few minutes, like the AchE inhibitors, as others like phosgene, kill after a latency period of hours, that is asymptomatic [23].

Incapacitating agents have the aim to rapidly cause disability, but not death. However, at great doses, these agents can also be lethal [23].

Harassing agents usually don’t act on vital organs and normally are a smaller threat compared to incapacitating agents. In this category riot control agents, as tear gases and malodorants are included. However, there is some controversy related to the classification of these agents as CWAs [23].

Since CWAs are extremely toxic and consequently, their use for investigation is limited, several simulant compounds are used to conduct studies. An ideal CWA simulant should replicate all the CWAs’ chemical and physical properties minus the toxic effects [24]. For example, in order to simulate mustard gases, compounds like 2-chloroethyl methyl sulfide (CEMS), 2-chloroethhyl phenyl sulfide (CEPS) and chloroethyl ethyl sulfide (CEES) were already used in several studies. In the case of nerve agents, namely tabun, sarin and soman other compounds are used such as, diphenyl chlorophosphate (DPCP), dimethyl methylphosphonate (DMMP) and diethyl ethylphosphonate (DEEPT). For VX, amiton (VG) is usually the chosen simulant agent [24].

### 2.2. Biological Warfare Agents: Incidents and Classification

In the matter of the use of BWAs, the plague is one of the most representative examples, since it was used in the World War II by Japan against China [3]. At this time, the consciousness of the threat of CWAs and BWAs was restricted to the military population. However, during the 1990s, due to the increasing number of terrorist attacks, the awareness of the civilian population about this problematic became more and more present, and is continually growing [5].

In December 2019 in Wuhan (Hubei province, China), several cases of pneumonia of unidentified origin were reported. These cases were epidemiologically connected to the Huanan Market, and after the analysis of several samples, a new respiratory coronavirus, SARS-CoV-2 was found. The cases related to SARS-CoV-2 disease (COVID-19) rapidly escalated, causing thousands of deaths all over the world. Regardless all the human lives lost, the severe economic repercussions lead to increasing poverty, with catastrophic consequences [25].

Other threats like the severe acute respiratory syndrome (SARS), avian flu and specially 9/11 showed the large variety of agents that can inflict severe repercussions in the world’s population. The 1984 salmonella food poisoning event by a cult in an Oregon restaurant led to no fatalities but several casualties, about 751, showing the huge impact of the BWAs threats [3]. Consequently, these events also demonstrated the importance of an “all-hazardous” strategy in order to improve the response of the responsible authorities [17].

In the specific case of the “anthrax letters”, it was shown that a weaponized version of bacteria could be used and sent, in this situation against elected official and the media, but also against the general population. The believe on an almost certain imminent CBRN attack led to the implementation of several initiatives and procedures for the defense against terrorism by several international entities, including the European Union (EU) [3,26].

BWAs are more lethal than the chemical ones, but they don’t have the capability to penetrate the skin, attaching themselves to clothing in high concentrations, resulting in large contaminations [18].

This type of agents can be described as a group of living biological organisms, that are used with the aim to create a state of war, by injuring or even causing death to humans, animals and plants. The BWAs include bacteria, viruses, fungi and insects. Whereas chemical weapons are used to kill living organisms, biological weapons use living organisms to kill [27].

These agents are mostly disseminated as aerosols in order to be more effortlessly distributed over a larger area. Nevertheless, some biological agents can be propagated from person to person, by ingestion, due to the contamination of food and water supplies, direct contact and by infectious vectors, like fleas or mosquitoes, that transmit the pathogen [28]. The main advantages in the use of BWAs are related to their easy and fast production, cost effectiveness, high mobility and morbidity, high person to person transmission and the fact that the consequences induced by this type of agents are difficult to treat [27].

Anthrax is considered the most effective BWA. This agent forms spores that resists to environmental activation. Although it’s not transmissible, affecting only its target, it causes an infection that escalates quickly with a fatality rate of more than 80%. Anthrax is one of the agents presented in the group of the most probable pathogens and toxins to be used as BWAs, denominated “Dirty Dozen” [28] (Table 1).

### 2.3. Chemical and Biological Protective Textiles

In the case of fibrous structures/textiles that are normally used for the development of protective clothing and facemasks, almost all of them are passive, having a permeable layer (usually activated carbon), which only acts as toxic agent’s adsorbent [10].

Considering PPE for chemical protection, the first generation of chemical suits was based on an inner layer of activated carbon fiber in a polyurethane foam and an outer layer with hydrophobicity properties. Most recently, in the United States the most used chemical suit is known as Joint Service Lightweight Integrated Suit Technology (JSLIST) and is based on beaded activated carbon as the inner layer of the knitted laminate and an outer layer of nylon and cotton. As the activated carbon presents several disadvantages, efforts are being made in order to reduce its quantity on JSLIST, namely by its replacement with perm-selective membranes, functionalized nanofibers and superhydrophobic materials [18].

The development of active fibrous systems able to decompose harmful agents, instead of passive ones is of great importance. These systems should be capable of self-decontamination as well as detection of toxic materials such as organophosphorus compounds (which are found in both pesticides and CWAs) [10].

Although effective, the methodologies that offer only passive protection, lead to a major problem, the surface of the equipment remains contaminated with toxic agents (because they are only adsorbed), requiring a decontamination process. Active protection systems do not require post-use decontamination and do not function only as adsorbents or as a skin barrier. Thus, this type of technology is preferable [30].

In the case of PPE for biological protection, biocidal coatings are presented as one of the best alternatives. N-halamine biocidal polymers can be used for several biocidal applications since they possess nitrogen-bromide or nitrogen-chlorine covalent chemical bonds. Another material with proven antimicrobial activity, which is essential for this application, are metal oxide NPs [18].

The PPE already available in the market need to be upgraded and reassessed, namely in the protection and user’s comfort properties. The last one is of huge importance, since for the soldiers in the extreme conditions of the battlefield the weight of the equipment is a crucial factor, that can affect their well-being, as well as their performance. So, the development of materials that present several properties, including protection against CWAs and BWAs without compromising the soldier’s comfort and by even improving it, is essential [14].

It’s also very important to have in mind some factors related to CBRN intoxication that influence the requirements of the PPE. The noxious effects of CBRN agents are dose dependent, and in a chemical weapon release scenario the first responders have the obligation to not become a victim themselves. Thus, the PPE need to be employed in order to protect the responders from an unique microenvironment with specific conditions, like its own temperature, humidity and odors [31].

Nowadays, the military suits used in CBRN scenarios are mainly based on activated charcoal, which sometimes is impregnated with metal ions. This material allows the physical adsorption of the CWAs and the filtration of the BWAs onto the equipment’s surface, preventing the contact of these agents with the wearer. Nevertheless, they present several disadvantages that are related to their moisture absorption, which directly diminishes the user’s comfort and adds weight to the equipment, and the decreasing absorption capability with time [32].

Textiles are usually the chosen substrate for PPE due to their versatility. This material can be constituted by one or a mixture of polymers and coatings (that can be coarse or fine, even up to nanoscale) and that can be assembled in a variety of structures. The biggest benefit in the use of textiles is their large contact with the user, without compromising the comfort. One of the other advantages in the use of this type of material is the wide knowledge and familiarity that the society has about their use and production. Thus, the use of textiles doesn’t need specific guidelines, and their production is already well established and developed [32].

Usually, fabrics made from synthetic fibers, namely polyethylene, polypropylene (PP), polyester (PES), polyamide and polyurethane are the most used for the production of protective clothing, since they represent a good balance between performance and cost. For example, polyamide fibers coated with polyurethane are a great alternative to high performance fibers, allowing a great abrasion resistance. Natural fibers, like cotton and wool are also frequently used for protection, but in this specific case for thermal protection and comfort properties. These fibers are often mixed with high performance fibers and usually need special treatments and finishing, such as coatings or lamination [33].

As referred before, CBRN terrorism is undoubtedly a rising threat worldwide. Thus, a solidly increase of the CBRN market is expected, especially regarding protective equipment, which will comprise most the CBRN market share in the next years. This increasing interest and development of protective equipment and technology should not only be used for military, but also by the civilian population [34].

The production of CBRN hazards, as well as the threat of terrorism utilizing weapons of mass destruction are in constant growth. In consequence, the development of suitable equipment for not only the protection, but also for the detection and analysis of harmful agents is becoming a lot more complex and difficult. Several factors, such as the appearance of new hazardous agents, economical limitations and strict safety and environmental legislations, difficult the development of PPE and need to be take into consideration [34].

## 3. Nanoparticles

NPs are one of the materials that is gaining more attention not only for the application on PPE for CWAs and BWAs, but also for the development of multifunctional systems. Due to the nanoscale dimension and very high surface area to volume ratio (SVR), nanomaterials present an enormous potential for imparting various functionalities to textiles. This multifunctionality could include: easy/self-cleaning, decomposition of chemical agents, antimicrobial activity, flame retardancy, UV protection, antistatic properties and monitoring/sensing behaviour [12]. The combination of metal oxide NPs with natural compounds can also be very interesting, especially for UV protection purposes. Chitosan and black plum peel extract were already used in combination with titanium dioxide (TiO_2_) NPs in order to improve their UV protection capability [35]. The combination of TiO_2_ NPs with cinnamates, presented in carnauba wax was also already studied, being verified that the introduction of the natural compound improved the sun protection factor (SPF) values, in this specific case of cosmetic formulations [36]. Zinc oxide (ZnO) NPs combined with aloe gel extract demonstrated better UV resistance properties [37]. *Scutellaria radix* (the root of *Scutellaria baicalensis Georgi*) was also combined with ZnO NPs, leading to an increase of the SPF values, in this situation for the formulation of sunscreen creams [38].

Carbon based nanomaterials, like carbon nanotubes and graphene are excellent examples for increasing fibers’ electrical properties but they are also effective in the decomposition of harmful chemicals [39,40,41]. Metal NPs, like silver (Ag), gold (Au) or copper (Cu) are commonly used in the development of antibacterial fibers [42,43,44]. Metal oxide NPs, namely: ZnO, TiO_2_, silica (SiO_2_), aluminum oxide (Al_2_O_3_), magnesium oxide (MgO) and cerium oxide (CeO_2_) can be used for UV protection purposes and decomposition of organic compounds, pesticides and microorganisms [45,46]. Within all the active functions, the degradation of chemical and biological harmful agents is currently considered a critical issue, not only for the military area but also for several other areas including healthcare, industry, agriculture and space [1].

Metallic NPs, metal oxide NPs and graphene oxide, in addition to the applications above, are being used in the adsorption and decomposition of harmful toxins.

### Metal Oxide Nanoparticles and Photocatalysis

**Figure 3:**
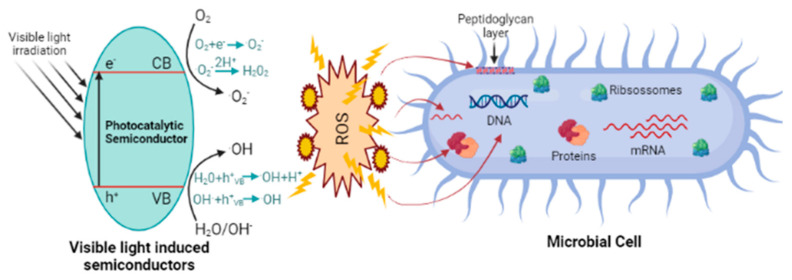
The possible mechanisms of antimicrobial activities exhibited by photocatalytic semiconductors in the right side of the figure and in the left side, the activation of the photocatalytic semiconductor by visible light. Created with BioRender.com.

Transition metal oxides present exceptional photocatalytic activity for the degradation of chemical agents, including organic compounds. Nanomaterials based on metal oxides with well-defined structural, crystalline and surface characteristics act like wide band gap semiconductors [6]. The photocatalytic mechanism consists of the absorption of UV radiation (by the semiconducting material) with energy higher than its’ band gap energy level. The electrons of the metal oxide’s surface absorb the incident light energy and are promoted from the valence band (VB) to the conduction band (CB), creating holes (h^+^) and generating electrons (e^−^). Then, these products react with adsorbed water and atmospheric oxygen producing reactive oxygen species (ROS), namely hydroxyl groups (•OH) and super oxide anions (•O_2_^−^), which can quickly initiate redox reactions with the organic compounds in their surroundings, leading to the formation of harmless byproducts. Thus, photocatalysis can be presented as a green and powerful way to degrade organic pollutants and to remove hazardous inorganic compounds [47] (Figure 3).

In the specific case of microorganisms, the ROS generated during photocatalysis are able to attack organic compounds presented in these organisms, namely cell membranes, RNA, DNA, proteins and lipids, resulting in their death [48] (Figure 3). Bacteria, not only Gram-positive but also Gram-negative, present negatively charged cell wall, which may influence the interactions between the bacteria and NPs, or the mostly positive ions released by them. For example, Gram-negative bacteria have a cell wall with a mosaic of anionic surfaces, instead of a continuous layer. Therefore, the binding of bigger quantities of NPs on these sites could increase the local toxicity [49,50].

Some examples of metal and metal oxide NPs for the decontamination and detection of warfare agents’ simulants, CWAs and BWAs were already reported (Table 2).

In literature, calcium oxide (CaO), MgO, TiO_2_ and Al_2_O_3_ NPs were already established as not only adsorbents, but also as being able of decontaminating toxic chemicals and CWAs [62,63,64,65]. Sensors with different chemical sensitive coatings of ZnO, tellurium dioxide (TeO_2_), Tin (IV) oxide (SnO_2_) and TiO_2_ were developed for the detection of warfare agents [51]. The capability of decontamination of CEPS (a sulphur mustard agent simulant) by ZnO NPs/Poly (vinyl alcohol) (PVA) surfaces was reported by Sadeghi et al. In this work, the NPs were synthetized by the sol-gel method, and the decontamination process was monitored by gas chromatography coupled to mass spectrometry (GC-MS) [52]. Neatu et al. demonstrated that Au/TiO_2_ systems, with Au NPs with 2–5 nm of diameter, were able to decompose the CWAs soman, VX and sulphur gas under visible light at room temperature. It was verified that 20 mg of TiO_2_ with 0.7 wt. % of Au was able to decompose 100 μL of dichloromethane with 0.77 wt. % of the toxic agent, after 2 h under visible radiation. At the end of the reaction, for all the warfare agents, the reaction products obtained were non-toxic [53]. The same research group reported the synthesis of TiO_2_ mesoporous materials with Au NPs and their successful influence as photocatalysts for the decontamination (under visible light) of the toxic agent soman [54]. Petrea et al. also studied the photocatalytic degradation of another CWA, sulphur mustard, using NiO-ZnO/TiO_2_ materials. This reaction was performed using 0.77 wt. % of the toxic agent, under UV radiation. For the best quantity of the nanocomposite, half of the initial quantity of the sulphur mustard was degraded in less than 20 min. These tests were made on several contaminated surfaces, confirming the activity and the stability of the developed materials [55].

Another example of the use of metal oxide NPs for CWA decontamination is the commercial product Fast-Act^®^, based on MgO and TiO_2,_ that is able to degrade soman and VX [56]. Wagner et al. developed self-decontamination paints for military vehicles based on TiO_2_ for the hydrolysis of VX, GD and HD [57]. The antibacterial activity of metal oxide NPs is also well-known [58]. ZnO and MgO have been proved to inhibit the growth of *Pseudomonas aeruginosa* and *Staphylococcus aureus* (*S. aureus*) [59], *Xanthomonas oryzae pv. oryzae* [60] and *Ralstonia solanacearum* [61].

The almost full decomposition of DMMP (a sarin nerve gas simulant) by a graphene oxide-MnO_2_ nanocomposite, prepared by thermal hydrolysis, was also demonstrated by Tolasz et al. The developed nanocomposites presented a better degradation ability when compared with pure MnO_2_ and graphene oxide. It was also confirmed that the quantity of graphene oxide affected the nanocomposites activity substantially [40]. The work performed by Sayago et al. also reported the use of graphene oxide nanomaterial as a sensitive layer for the detection of DMMP and DPGME (a simulant of nitrogen mustard). The developed sensor exhibited outstanding sensitivity, good linearity and repeatability to all the tested simulants [41]. It’s important to notice that all the selected studies, which report the decontamination of CWAs and their simulants, only evaluated the activity of the isolated NPs and NPs with polymers.

## 4. Fibrous Structures Functionalized with Nanomaterials

The development of fibrous systems functionalized with NPs for chemical/biological decontamination has been poorly explored. However, some work was already performed exhibiting promising results, specially involving the development of multifunctional structures, which could be excellent candidates to be used in PPE. In the following sections, several examples related with textile structures functionalized with (1) metal and metal oxide NPs, (2) carbon nanomaterials and (3) metal-organic frameworks (MOFs) will be summarized. In all the examples special attention will be given to the ones exhibiting the use of natural fibers.

According to literature, there are some examples of chemical/biological decontamination systems using synthetic fibers, polymers, nanocomposites and electrospun nanofibers functionalized with NPs [66,67,68]. However, the use of natural fibers and the in-situ functionalization of the NPs onto these fibers is a very promising area that is yet to be thoroughly explored. Although these materials may not be used alone for this type of applications, they can be a great complement, or can even be used in combination with other materials.

Natural fibers can be divided in two groups depending on their origin (animal or plant). These materials, such as: flax, jute, hemp, cotton, silk and wool can be collected directly from nature and are considered environmentally friendly materials. Due to their low cost, light-weight, abundance and biodegradability they are undoubtedly promising materials for replacing the synthetic ones, even if only in a relative percentage [69]. Besides all of these great properties, these fibers are easily functionalized, being a very interesting material to be used as substrate for PPE applications [70].

Electrospun nanofibers embedded with NPs present great filtration properties and are excellent candidates for the replacement of material such as glass fiber and activated charcoal [71]. These nanofibers are one of the future trends in the development of fibrous structures for PPE applications. Hence, and in order to finalize this literature review, not only their potential for the detection/decontamination of CWAs and BWAs, but also for the development of multifunctional fibrous materials will be presented.

### 4.1. Metal and Metal Oxides

Grandcolas et al. developed self-decontaminating layer-by-layer functionalized textiles (cotton/polyamide (50/50) military textiles), with a layer of a dense network of entangled tungsten trioxide (WO_3_) and modified titanate nanotubes. These systems were able to degrade DMMP under solar light illumination and the blister organosulfide yperite live chemical weapon agent [72].

Despite the shortage of work on textiles functionalized with NPs for the decontamination of CWAs and their simulants, the functionalization of fabrics with metal oxide NPs in order to obtain fibrous systems with several properties has already been reported. Duan et al. reported the functionalization of cotton fabrics with CeO_2_ sol and modification with a layer of dodecafluoroheptyl-propyl-trimethoxylsilane (DFTMS). They obtained not only superhydrophobicity, reaching a water contact angle (WCA) of almost 158°, but also excellent protection against UV radiation [46].

A protective textile was developed using N-doped TiO_2_ embedded citral microcapsule coating. The coated textile presented photocatalytic activity for the degradation of formaldehyde under visible light irradiation. These materials could be used for air purification applications [73].

The development of a multifunctional textile based on polyester modified with natural polysaccharide alginate and colloidal TiO_2_ NPs was described by Mihailovic´ et al. The functionalized fabrics presented great antibacterial activity against *Escherichia coli* (*E. coli)*, UV protection efficiency and total photodegradation of methylene blue, reached after 24 h of UV irradiation. The authors also reported the durability of the modified textiles after 5 washing cycles [74].

The same investigation group described the development of multifunctional PES fabrics with colloidal Ag and TiO_2_ NPs. Once again, they obtained the total photodegradation of methylene blue, after 24 h of UV irradiation, UV protection (Table 3), and antimicrobial activity against Gram-negative bacteria *E. coli*, Gram-positive bacteria *S. aureus*, and fungus *Candida albicans* [75].

Abdelrahman et al. developed multifunctional printed textiles based on three metal oxide nanoparticles, TiO_2_, MgO and ZnO. Natural (wool) and synthetic (acrylic) fibers were pretreated with the different metal oxide nanoparticles, followed by printing using a past based on polylactic acid. Another method was tested, by applying the metal oxides after the printing process. The addition of the nanoparticles onto the fabrics surface resulted in an increase of the color strength. Additionally, the developed materials showed good antibacterial activity, high UV protection and self-cleaning activity (decomposing methylene blue) [76].

The functionalization of 100% cotton fabrics by in-situ synthesis of nano-ZnO was developed by Prasad et al. The fabrics maintained the UV protection factor (UPF) above the minimum accepted level of 50 till 50 wash cycles. Excellent antibacterial activity (>98%) against two representative pathogens, *S. aureus* (Gram-positive) and *Klebsiella pneumoniae* (Gram-negative) was also obtained, even after 50 wash cycles [77].

Self-cleaning cotton fabrics were developed by the functionalization of cotton with photocatalytic ZnO NPs. Methylene blue was used as a test contaminant to evaluate the self-cleaning properties of the modified fabrics. The authors verified that this property was dependent of the NPs concentration. They also exhibited great UV blocking properties [78].

**Figure 4:**
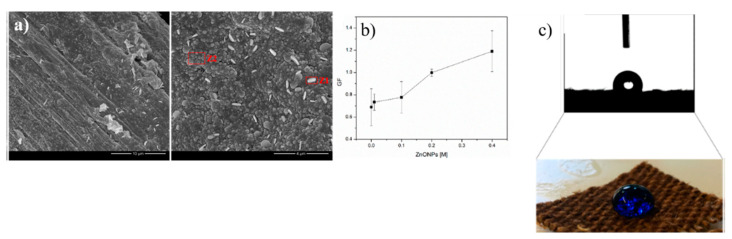
FESEM images of flax fabric functionalized with both Ag and ZnO NPs (**a**). Gauge Factor (GF) values for the flax fabrics functionalized with different ZnO NPs concentrations (**b**). WCA measurements of the flax fabrics functionalized with Ag and ZnO NPs (**c**) [79].

Multifunctional flax fibers based on the synergistic effects of Ag and ZnO NPs were developed by Costa et al. (Figure 4). The developed samples exhibited piezoresistive response and the sensor sensitivity increased with the use of higher ZnO precursor concentration. Great antibacterial activity values against Gram-negative and Gram-positive bacteria and high WCA values, higher than 100°, were also obtained. The fibrous systems also presented wash durability and UV radiation resistance. Thus, the developed multifunctional natural fibrous systems can be applied in a wide range of monitoring/sensing applications, especially as piezoresistive sensors. The antibacterial activity as well as the hydrophobic character can also be very beneficial in several areas, including in the decontamination of noxious agents [79].

Pereira et al. developed fibrous structures based on flax and CaO NPs with degradation properties and antibacterial activity. The influence of the surface’s pre-treatments was studied, in order to infer their influence on the anchorage of the NPs. Alkali, Acetylation and Potassium Permanganate were tested. The flax fabrics treated using the alkali method obtained the best results, hence these fabrics were the ones used for the rest of the work. The efficiency of the developed samples as systems for the protection against CWAs and BWAs was evaluated, testing the degradation, under UV radiation, of DMMP and the antibacterial activity against *S. aureus* and *E. coli*. DMMP was successfully degraded. However, the antibacterial results were not the most promising for the conditions that were used [80].

**Figure 5:**
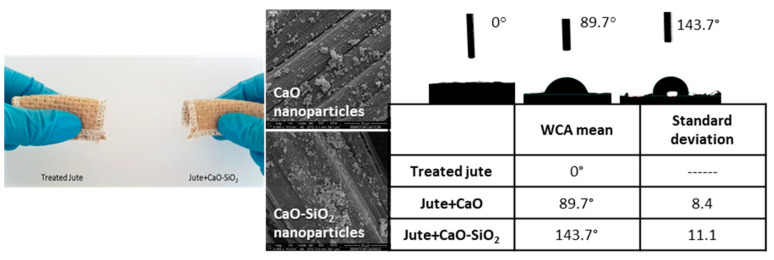
Digital photo and FESEM images of the jute fabrics functionalized with CaO and CaO-SiO_2_ NPs and WCA values. Used with permission from [13].

Araújo et al. developed a multifunctional fibrous system based on jute fibers and CaO and SiO_2_ NPs with antibacterial activity, UV protection, hydrophobicity, methylene blue degradation and antibacterial activity (Figure 5). The developed samples presented very good WCA values, reaching 143.7° and UPF values of 50+ for the functionalized fabrics. The obtained antibacterial activity values were of 99.96% against *S. aureus* and 99.80% against *E. coli*. Regarding the methylene blue degradation, the samples were exposed to UV radiation, and after 24 h the characteristic methylene blue adsorption band almost disappeared, indicating that the dye was degraded. These results confirm the potential of the developed systems for the application in PPE, namely for the protection against noxious chemical and biological agents, since they present the capability to degrade a noxious dye, which can possibly be translated in the degradation of CWAs, and since they present antibacterial activity against Gram-negative and Gram-positive bacteria, which can be very beneficial for the decomposition of BWAs. The wash durability of the developed fibrous systems was evaluated, demonstrating that the NPs were well anchored to the jute fabrics [13].

**Figure 6:**
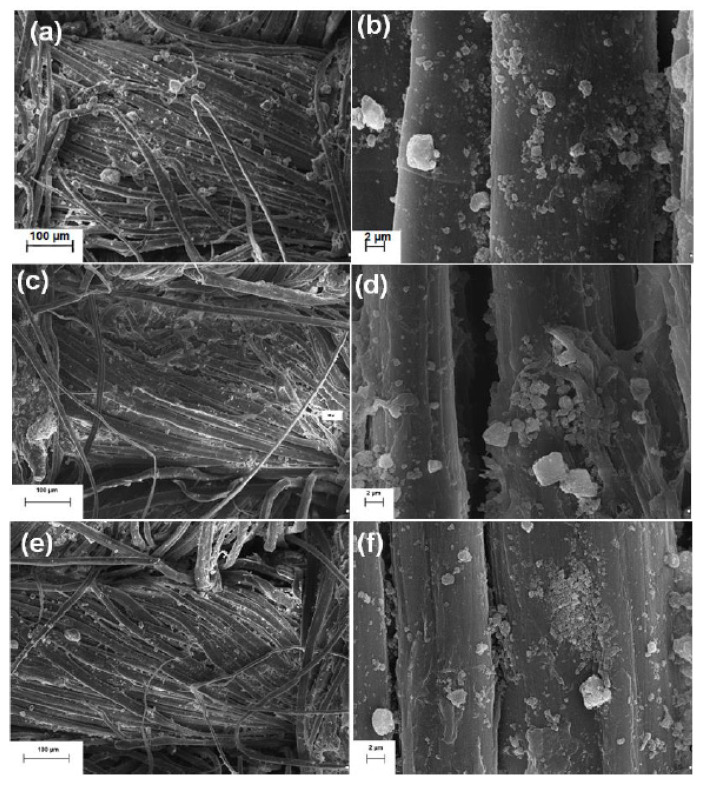
SEM images of: (**a**,**b**) linen fabric after alkaline treatment and dip-coating with 5% wt. of CuO-SiO_2_, (**c**,**d**) linen fabric after enzymatic treatment with 2.5 U/g of laccase and dip-coating with 5% wt. of CuO-SiO_2_, (**e**,**f**) linen fabric after enzymatic treatment with 5.0 U/g of laccase and dip-coating with 5% wt. of CuO-SiO_2_ [11].

Olczyk et al. compared and studied the influence of two different linen pretreatments, alkaline and enzymatic. The chosen treatment was a combination between a bio-pretreatment (using laccase from *Cerrena unicolor*) and modification with CuO-SiO_2_ hybrid oxide microparticles (dip-coating) (Figure 6). The final samples presented great antimicrobial activity against *Candida albicans* and against Gram-positive (*S. aureus*) and Gram-negative (*E. coli*) bacteria. The UV protection capacity obtained was also very interesting, with UPF values of >40. The developed samples showed special potential to be used as clothing or outdoor textiles, since they offer protection against microorganisms and UV radiation [11].

Vasantharaj et al. used an aqueous extract of *R. tuberosa* for the synthesis of copper oxide (CuO) NPs, that were used to functionalize cotton fabrics. The developed fabrics presented bactericidal activity and photocatalytic degradation of crystal violet dye, under direct sunlight [81].

Attia et al. developed a coating based on SiO_2_ NPs (derived from waste agriculture rice husk) and Ag NPs, that were immobilized on the SiO_2_ NPs’ surface, and applied on the surface of a cotton/polyester blend fabric. The treated fabrics presented antibacterial activity, UV protection capability and hydrophobicity, with a WCA of 145° [82].

Multifunctional silk fabrics were developed by Gao et al. by the coating of the silk’s surface with nano-SiO_2_. Regarding the UV protection, the highest UPF value reached for the coated fabrics was of 84.52. The developed samples also showed not only improved wrinkle resistance, but also hydrophobicity. It was also proved that the nano-SiO_2_ particles were well attached to the fabric’s surface. The materials also demonstrated great thermostability and cytocompatibility, as well as laundry resistance. Therefore, the multifunctional silk fabrics demonstrated potential to be used for self-cleaning, protection and non-ironing applications [83].

Ferreira et al. developed a multifunctional fibrous system based on natural fibers functionalized with Ag NPs, with special focus on the electrical conductivity and antibacterial activity. Several natural fibers, such as flax, jute, sisal, coir and cotton were successfully functionalized using a very sustainable method, polyethylene glycol (PEG) reduction. The resistivity of the functionalized fabrics was of 1.0 × 10^3^ Ω·m and they presented antibacterial activity against *S. aureus* and *E. coli*. The developed samples can not only be used for sensing/monitoring military applications, but also for the protection against bacteria [84].

**Figure 7:**
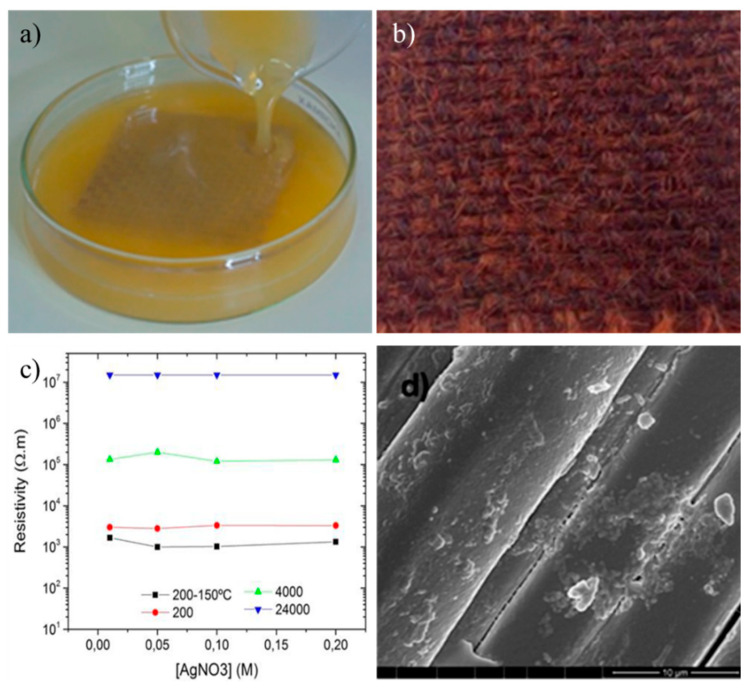
Jute fabric impregnated with Ag^0^-PEG suspension (**a**). Jute fabric with Ag^0^-PEG NPs (**b**). Dependence of the resistivity values on AgNO_3_ concentration (**c**) FESEM images of jute fabrics functionalized with Ag NPs (**d**) [85].

The same research group developed conductive fibrous structures based on jute fibers and Ag NPs using a green sustainable approach (Figure 7). The Ag NPs were applied onto the jute fabrics using two different and sustainable strategies, UV photoreduction and using PEG as the reducing agent and stabilizer. The resistivity value obtained for the non-functionalized jute fabric was of 1.5 × 10^7^ Ω·m, however, for the functionalized fabrics it decreased almost 15,000 times, reaching 10 × 10^3^ Ω·m. Therefore, the obtained conductivity was of 0.001 S/m, which is a considerable value for a functionalized natural fiber [85].

Zhou et al. once again functionalized silk fabrics, this time with Ag NPs, in order to obtain a multifunctional textile. The Ag NPs were synthetized employing an eco-friendly method, using a natural extract as the reductant and stabilizing agent. The developed materials demonstrated antibacterial activity against *E. coli* and *S. aureus* and antioxidant activity. Overall, this work demonstrated a sustainable way to synthesize Ag NPs, as well as the obtention of an ecological material that can be used for medical applications [86].

A multifunctional silk textile was modified with TiO_2_ and TiO_2_@Ag NPs by Li et al. Especially the samples with TiO_2_@Ag demonstrated great UV protection capability and antibacterial activity, namely against *E. coli*, *S. aureus* and *Pseudomonas aeruginosa*. The degradation of a dye, methylene orange, under UV illumination was also tested. The developed material presented strong photocatalytic activity and self-cleaning properties [87]. Rehan et al. incorporated Ag NPs by the in-situ method in natural fabrics, cotton and wool. The successful incorporation of the NPs was confirmed by several characterization techniques. The developed samples demonstrated multifunctionality, namely colorant, antibacterial activity and UV protection [88].

Cotton fabrics with an Ag NPs finishing were produced by Islam et al., being that the NPs were synthetized using a green biochemical reduction method. Pomegranate peel extract was used as the reducing and capping agent for the Ag synthesis. Not only the NPs effective production but also their deposition onto the cotton fabrics’ surface were confirmed by different characterization techniques. The coloration effect of the Ag NPs was evaluated, and it was verified that they imparted a pale yellow color to the initial brown cotton. Very good antioxidant and antibacterial activity against *E. coli* and *S. aureus* were also verified [89].

Shabbir et al. synthetized Ag NPs by the in-situ method using natural compounds as reducing and stabilizing agents, and then deposited them onto wool fabrics. The introduction of the NPs allowed the coloration of the cotton fabrics, with different color shades, depending on the morphology of the particles. Great values of antioxidant activity of the developed samples were obtained, as well as very good UV protection performance [90].

Nanocomposite cellulose fibers were functionalized in-situ with Ag NPs by Rac-Rumijowska et al. The fibers were produced using an environmentally friendly method and the doping with the NPs was made by a direct and in-situ reduction of Ag^+^ ions. The developed samples demonstrated great antimicrobial activity against not only *S. aureus* and *E. coli*, but also against *Acinetobacter baumannii* and *Candida albicans*. The addition of the Ag NPs also reduced the heat release of the fibers by 36 %, as proven by the flammability evaluation. The electrical properties were also evaluated, and for the fibers with 3 wt. % of Ag, the linear resistance was of 10^8^ Ω/cm [91].

Rehan et al. reported the development of multifunctional cotton fabrics functionalized with Ag NPs. The samples were produced by the in-situ synthesis of Ag NPs onto the cotton fabrics, using a green UV-reduction method. The addition of the Ag NPs imparted coloration to the fabrics with a wide range of shades. The developed samples also presented antibacterial activity against *E. coli* and UV protection [92]. Another example of multifunctional cotton fabrics is the work of El-Naggar et al., that reports the in-situ preparation of Ag NPs onto the fabrics by the pad-dry-cure method. The developed technical textiles demonstrated antimicrobial activity, namely against *S. aureus*, *E. coli* and *Candida albicans* and self-cleaning capability, since they were able to decompose methylene blue [93].

Cotton fabrics were functionalized with Au NPs by Tang et al. The NPs imparted color to the fabrics, presenting good colorfastness to washing and rubbing. The functionalization improved the UV protection capability of the cotton fabrics and imparted antibacterial activity [94].

### 4.2. Metal Organic Frameworks (MOFs)

Lee et al. reported for the first time the photocatalytic activity of an Al-MOF (Al porphyrin MOF), based on an earth-abundant metal-containing Al(OH)O_4_ cluster bridge by H_2_TCPP (5,10,15,20-tetrakis(4-carboxyphenil) porphyrin) chromophores. The MOF was immobilized into polymeric fibers and it was verified that the developed samples could detoxify a sulfur mustard gas simulant, CEES under visible-light radiation [95].

Jung et al. reported the development of an omniphobic textile surface, able to repel not only water but also CWAs. The structures were based on a Zirconium (Zr) porous MOF and on a polyhedral oligomeric silesquioxane (POSS), in order to control the surface’s structure [96]. A easy method to assemble presynthesized UiO-66-NH_2_ crystals onto nonwoven PP fibrous mats was developed by Lee et al. These materials were able to degrade a CWA simulant, dimethyl 4-nitrophenyl phosphate (DMNP) with a half-life of <5 min [97].

The fabrication of multifunctional e-textiles by the incorporation of two-dimensional (2D) MOFs into fabrics was explored by Smith and Mirica. These materials presented good wash durability as well as high porosity and flexibility. The fabrics were able to detect different gas analytes as well as to capture and filter them [98]. Emam et al., developed anti-UV radiation textiles by incorporating nano-MOFs (MIL-68(In)-NH_2_ and MIL-125(Ti)-NH_2_) into silk and cotton. The successful formation of the MOFs was confirmed by electron microscopy and X-ray diffraction. The functionalized fabrics presented a great UV protection capacity (Table 4), even after some wash cycles [99].

Bunge et al. functionalized cotton fabrics with UiO-66-NH_2_ MOFs. These materials were able to react with DMNP, which was monitored by UV-vis spectroscopy. This work demonstrated that MOF-natural composite can present a similar performance, regarding CWAs simulants reactivity, as synthetic ones [100].

Cotton fabrics with Cu-BTC MOF and oxidized graphitic carbon nitride, g-C3N4-ox were developed by Giannakoudakis et al. The functionalized fabrics demonstrated the ability to detoxify a nerve gas substitute, dimethyl chlorophosphate. The developed fabrics also changed colors after the decontamination process, which demonstrates their ability to be used for detection applications. These materials were able to adsorb 7 g of the simulant per gram of Cu [101].

Protective textiles that can be applied in everyday life, in this case for the protection against UV radiation and noise mitigation were developed by Zhang et al. The protective textiles were prepared by the modification of cotton fabrics with MOFs nanocrystals. The introduction of the MOFs improved the UV blocking capability as well as the acoustic absorption, when compared with neat cotton. The successful functionalization with the MOFs was confirmed using several characterization techniques. The work of Zhang et al. demonstrated the preparation of a multifunctional protective textile, using natural fibers as the substrate [102]. Jhinjer et al. functionalized cotton fabrics by the in-situ synthesis of zeolitic imidazole framework (ZIF-8 and ZIF-67) MOFs. Additionally, the sustainability of the used methods was taken into consideration. The strong anchorage of the ZIF nanocrystals onto the fabrics’ surface was proven by several techniques. The developed samples demonstrated great adsorption capacity, namely of organic pollutants like aniline, benzene and styrene in high quantities. These materials also shown that they could be reused and regenerated, without losing their efficiency. Therefore, the produced fabrics showed a great potential to be use as protective textiles [103].

### 4.3. Carbon Nanomaterials

Medical grade polyviscose textiles pads were functionalized with Ag NPs and reduced graphene oxide by Noor et al. using a surface-mediated wet chemical solution-dipping process. The developed samples demonstrated antibacterial activity against *E. coli*, being that after 12 laundering cycles, this activity only had a small decrease (from 100% to 90.1%). The authors also verified that the graphene oxide improved the anchorage of the Ag NPs onto the fabrics [104]. Jin et al. studied the potential of functionalized graphene (FG) as reinforcement to produce nylon 11 and 12 nanocomposites. The functionalization with only a small amount the FG led to a significant improvement of the mechanical properties of the nylon, namely the tensile strength, elongation at break, impact strength and toughness. These functionalized materials also presented very good vapor and gas barrier properties, once again with only a small FG loading. For the nylon 11, 0.3 wt. % of FG was enough to reduce the water vapor and oxygen permeability by ~49 and ~47%, respectively [105].

Cotton fabrics were impregnated with reduced graphene oxide coated Cu and Ag NPs by Bhattacharjee et al. The functionalized fabrics demonstrated washing durability since the graphene surface-cotton fabric and the NPs were strongly connected. After the functionalization, the hydrophobicity of the cotton fabrics increased, as well as the UV protection capability and the thermal stability [106].

Cai et al. also functionalized cotton fabrics, but this time with only graphene oxide, which was reduced by a simple in-situ method. The graphene oxide coating was applied by an easy “dip and dry” process. The developed samples demonstrated not only good electrical conductivity but also hydrophobicity and UV protection capability. These properties were maintained after washing and after repeated manipulation of the samples [107]. Cotton fabrics functionalized with graphene oxide decorated with Fe, N-doped TiO_2_ NPs were presented by Stan et al. Their photocatalytic effect was tested, and it was concluded that with higher quantities of TiO_2,_ the higher this effect under visible light. The samples also presented hydrophobicity, successively increasing with the increase of the graphene oxide layers. Resistance against *Enterococcus faecalis* and *E. coli* was also registered [108].

Silk fabrics with graphene oxide with multifunctional properties, namely electrical conductivity, UV protection and water repellence were produced by Cai et al. The graphene oxide deposited onto the silk fabric surface was reduced by sodium hydrosulfite. A low resistance value, of 3.24 KΩ cm^−1^ was obtained for the samples that were modified with graphene oxide 9 times. The UPF values of the silk fabrics also increased for the modified fabrics, as well as the hydrophobicity. In addition, the developed samples also demonstrated great wash durability [109].

Shirgholami et al. treated wool fabrics with a graphene/TiO_2_ nanocomposite. The nanocomposite was homogeneously distributed all over the wool fibers surface, as it was proven by several techniques. The developed samples were able to photocatalytic degrade methylene blue under sunlight radiation and also presented antibacterial activity. Additionally, the introduction of the nanocomposite also reduced the electrical resistivity of the fabrics and improved the UV radiation protection ability, without any negative impacts on their cytotoxicity [110].

**Figure 8:**
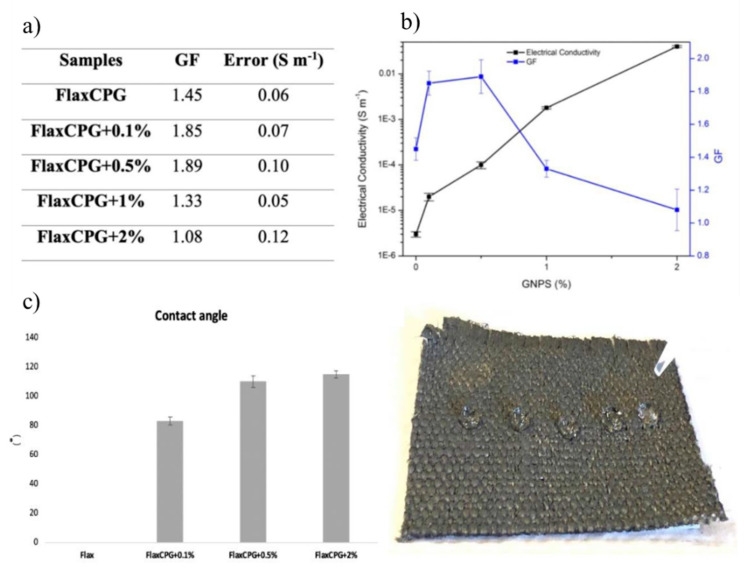
Average GF values (**a**) and relation between electrical conductivity and GF (**b**). WCA values obtained for the flax sample and the flax samples functionalized with the polymeric formulation with different concentrations of GNPs (**c**) [111].

Pereira et al. functionalized natural fabrics (flax) with chitosan based polymeric formulations of graphene nanoplatelets (GNPs) in order to develop a multifunctional ecomposite with electrical properties. Values of 0.04 Sm^−1^ of electrical conductivity were obtained for the sample with 2% of GNPs. A piezoresistive behavior was also observed, being that a GF value of 1.89 was obtained using 0.5% of graphene nanoplatelets. The developed samples also presented UV protection ability, with UPF values of 50+, and also hydrophobicity, reaching a WCA of 115° (Figure 8). This type of systems can be used for pressure sensing applications and to detect high loading, such as differences in human motion. The hydrophobic behavior and UV protection capability can also be very useful for other type of applications [111].

### 4.4. Nanofibers Produced by Electrospinning: New Trend

Electrospun nanofibers have also become one of the most advantageous structures to be use in protective textiles for chemical and biological defense [112]. The development of nanofibers with catalytic activity, capable of degrading CWAs/BWAs and/or capable of acting as sensors, with the ability to detect the adsorption of these agents, has been widely studied. In fact, Kim et al. fabricated zirconium (IV) hydroxide (Zr(OH)_4_)-coated nylon-6,6 nanofiber mats for the decontamination of chemical warfare nerve agents simulants. The developed composite nanofibers showed high decontamination efficiency against diisopropylfluorophosphate, a nerve agent analogue [113].

For the degradation of biological agents, some work has been made regarding the development of antimicrobial electrospun nanofibers, using polyamide, PVA, polyurethane, polypropylene, nylon-6, polyvinyl chloride and cellulose acetate, with the combination with several NPs, such as Ag, TiO_2_, Cu, ZnO, MgO and zirconium dioxide (ZrO_2_). These structures have shown various properties, like flame retardance, UV protection, self-cleaning and antistatic activity [114].

**Figure 9:**
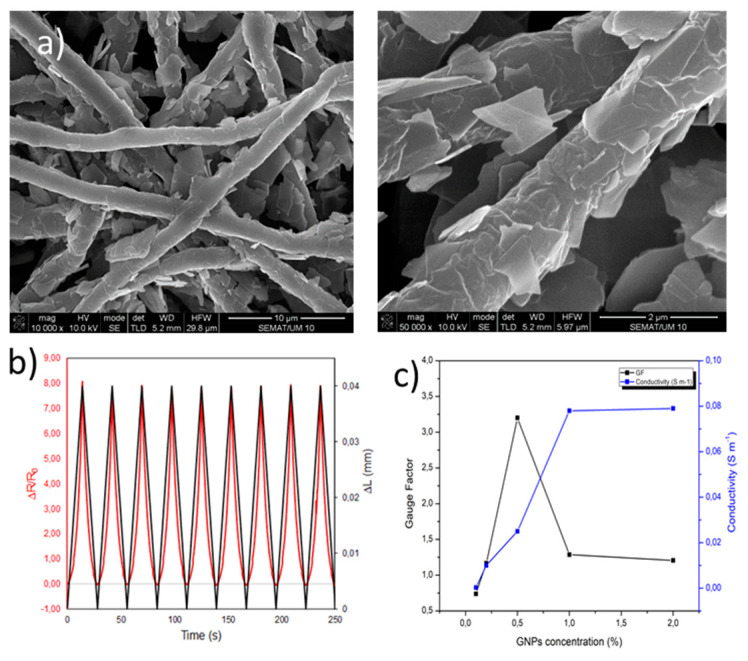
FESEM images of PCL microfibers with GNPs (**a**). Piezoresistive response of GNPs-PCL membrane (**b**). Electrical conductivity and GF in relation to the GNPs concentration of GNPs-PCL membrane (**c**) [115].

Francavilla et al. developed electrospun microfibers of polycaprolactone (PCL) functionalized with GNPs in order to produce a smart fibrous structure. A maximum value of electrical conductivity, 0.079 S/m, was obtained for 2% of graphene nanoplatelets. The developed samples demonstrated potential to be used as piezoresistive sensors, since they presented high sensitivity to external pressures and high durability to repetitive pressing. The best value of GF obtained was of 3.20 for the sample with 0.5% of GNPs (Figure 9). Therefore, the developed smart fibrous structures presented a great potential to be used as a wearable sensor, that can be applied on the monitoring of human motions and vital signs for soldier’s equipment applications [115].

**Figure 10:**
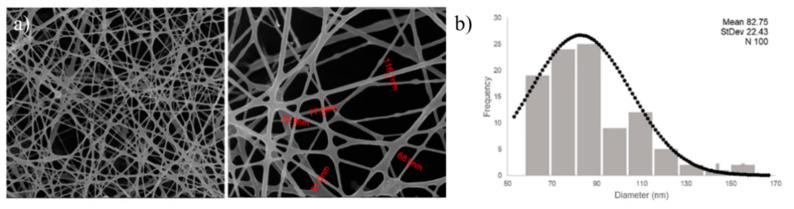
FESEM images of the chitosan, poly(ethylene) oxide, cellulose nanocrystals nanofibers incorporated with acacia extract (**a**). Diameter distribution histogram (**b**). Used with permission from [116].

Electrospun nanofibers based on chitosan, poly(ethylene oxide), cellulose nanocrystals and acacia plant-based extract were developed by Ribeiro et al. (Figure 10). The developed samples demonstrated antibacterial activity against 6 bacteria species, Gram-positive *Bacillus cereus*, *Listeria monocytogenes* and *S. aureus*, as well as Gram-negative *Enterobacter cloacae*, *E. coli* and *Salmonella Typhimurium*. They also demonstrated antifungal activity against 6 fungi as well as biocompatibility and continuous release of the natural extract during 24 h [116].

The same investigation group developed natural and biodegradable electrospun nanofibers using gelatin, chitosan, cellulose nanocrystals and natural propolis extract. Once again, the developed membranes demonstrated antibacterial activity against both Gram-positive (*S. aureus*) and Gram-negative (*E. coli*) bacteria, having the potential to be used in materials with bactericidal activity [117].

## 5. Conclusions

Harmful threats for the world’s population lives and well-being are constantly increasing and becoming more sophisticated and lethal. A wide range of CWAs and BWAs are already developed and their mechanism is optimized in order to originate the most harm possible. New agents are being created as we speak, since the use of these agents as weapons of mass destruction are more and more a threat. The COVID-19 pandemic is the perfect example of the severe consequences that a biological agent, in this case a virus, can cause on the world’s society, economy, and most important public health. Being that, most probably, this specific case wasn’t the consequence of a deliberate release, if the release of a harmful agent was well planned and focused, the consequences would be catastrophic. Having in consideration the CBRN incidents that have happened all over history, it’s essential to improve not only the awareness of governments worldwide about this topic, but also the protection of the military (in war zones) and the civil population, since we are more and more exposed to this type of hazards.

Several types of PPE were already developed and are now being used. However, a constant effort is being made in order to improve their performance. An essential factor for this optimization, is the type of protection. An active protection is preferred when compared to a passive one, since it allows the total degradation of the hazards, and doesn’t require a post-decontamination process.

The development of active fibrous structures with NPs, especially metal oxides, is undoubtedly a great strategy for the development of PPE with active protection. Besides the inherent characteristics of the nanoscale, metal oxide NPs present other properties that make them great candidates to be used in the adsorption and decomposition of harmful agents. The functionalization of fibrous substrates with NPs is an excellent way to guarantee a wide range of properties without adding the extra weight, essential for the user’s comfort.

In literature there are several reports on the use of metal oxide and metallic NPs, carbon nanomaterials and MOFs for the degradation of CWAs and BWAs. There are also some examples of fibrous systems functionalized with these NPs for the same type of applications. However, little has been done in order to improve the sustainability of the materials and processes used. Therefore, work needs to be done in to achieve the simplest and environmentally friendly PPE. The use of natural fibers can be a great strategy and an excellent alternative to the use of synthetic ones, due to their high abundance in nature, low cost and biodegradability. The use of simple and greener methods is also preferred.

## Figures and Tables

**Table 1:** Most probable pathogens and toxins to be used as BWAs (“Dirty Dozen”). Adapted with permission from [29].

Bacteria	Viruses	Toxins
Anthrax (*Bacillus anthracis*) Plague (*Yersinia pestis*) Tularemia (*Francisella tularensis*) Glanders (*Burkholderia mallei*) and meliodosis (*Burkholderia pseudomallei*) Brucellosis (*Brucella sp.*) Q fever (*Coxiella burnetii*)	Smallpox (*Variola major*) Viral encephalitis (e.g., VEE) Viral hemorrhagic fevers (e.g., Ebola, Marburg disease)	Botulinum toxin Ricin *Staphylococcal enterotoxin B*

**Table 2:** Literature examples of the use of metal and metal oxide NPs for the decontamination/detection of warfare agents/simulants [40,41,51,52,53,54,55,56,57,58,59,60].

Nanoparticles	Application	Warfare Agents or Simulants	Reference
ZnO, TeO_2_, SnO_2_ and TiO_2_	Detection	DMMP, DBS, CEPS and DECP	[51]
ZnO	Decontamination	CEPS	[52]
Au/TiO_2_	Decontamination	Soman, VX and sulfur mustard	[53]
Mesoporous TiO_2_/Au	Decontamination	Soman	[54]
NiO-ZnO/TiO_2_	Decontamination	Sulfur mustard	[55]
Fast-Act^®^ (MgO and TiO_2_)	Decontamination	Soman and VX	[56]
Self-decontamination paints (TiO_2_)	Decontamination	VX, GD and HD	[57]
ZnO and MgO	Antimicrobial activity	*Pseudomonas aeruginosa* and *Staphylococcus aureus*, *Xanthomonas oryzae pv. oryzae* and *Ralstonia solanacearum*	[59,60,61]
Graphene oxide-MnO_2_	Decontamination	DMMP	[40]
Graphene oxide	Detection	DMMP and DPGME	[41]

**Table 3:** UPF values of PES fabrics loaded with Ag and TiO_2_ NPs. Used with permission from [75].

Sample	UPF Value	UPF Rating
PES	43.0	40
PES + TiO_2_	91.6	50+
PES + Ag10 + TiO_2_	91.6	50+
PES + TiO_2_ + Ag10	76.1	50+
PES + Ag50 + TiO_2_	118.6	50+
PES + TiO_2_ + Ag50	112.0	50+

**Table 4:** UV blocking data for some of the developed anti-UV radiation textiles. Adapted from [99].

Samples	UVA (T%)	UVB (T%)	Blocking UVA	Blocking UVB	UPF	UPF Rate
Silk	17 ± 1.8	6.3 ± 1.2	83 ± 1.8	93.7 ± 2.1	10.5 ± 1.8	Insufficient
Silk/Ti-MIL-2	0	0	100	100	>100	Excellent
Cotton	34.3 ± 2.6	24.3 ± 2.1	65.7 ± 2.8	75.7 ± 2.3	3.5 ± 0.7	Insufficient
Cotton/Ti-MIL-2	0	0	100	100	>100	Excellent
